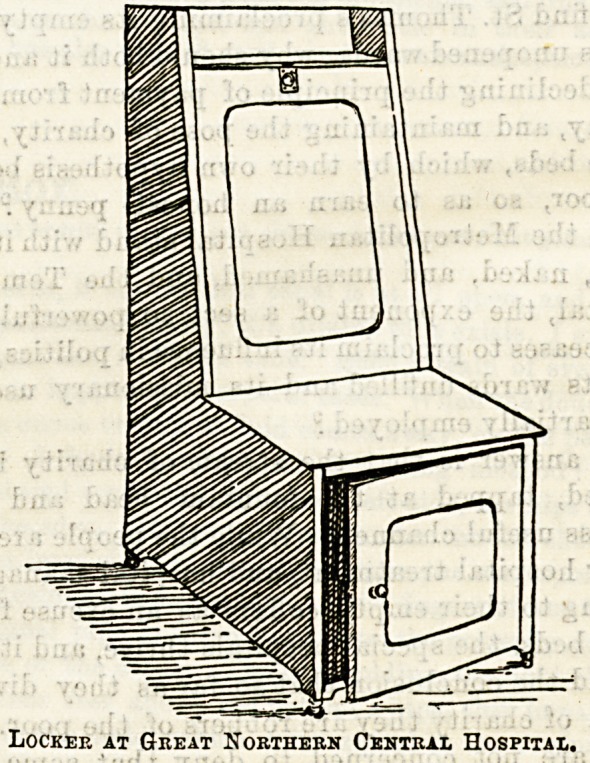# The Hospital Locker

**Published:** 1895-02-23

**Authors:** 


### PRACTICAL DEPARTMENTS.

### THE HOSPITAL LOCKER.

The comfort of a patient in the general wards of a large hospital may be very much increased or the reverse according to the capabilities of the "locker" which stands beside his bed, and which in most cases has to contain all the special private possessions which he is allowed to retain, and generally also such small stores as butter and sugar. The modern hospital locker is a vast improvement upon what originally answered to the name, its shape, size, and general construction will be found to differ in almost every hospital.

The fashion of using marble or glass for the tops has gained much favour, and is of course infinitely more hygienic than wood—even hard polished wood. Glass is better than marble, being impervious to the action of acids. For the body a hard wood, such as teak or oak, preferably teak, should be used, its polished surface offering less harbour for the insidious microbe. It is well that there should be certain divisions of space, to prevent the accumulation of too many divers articles together. The illustration we give above of a locker at the Middlesex Hospital shows a good disposal of the available space. The drawer at the top is for brushes and combs; the middle and largest space is devoted to stores, while the

LOCKER AT GREAT NORTHERN CENTRAL HOSPITAL.

lower little cupboard may be kept for any small private possession, books or papers, &c. On the upper shelf medicines are kept, and in this particular locker there is a place for temperature chart, &c., above. A towel rail is provided at the back. These Middlesex lockers are made of teak, with grey marble tops, and are made by the hospital carpenter.

Ventilation is effected by means of perforated zinc at the back.

Ventilation is an important point for consideration in a locker. A good plan is followed by Messrs. Atkinson, the well-known firm of furniture makers in Westminster Bridge Road, who supply so many hospitals with ward fittings, and that is to construct the back of the locker of lathes sufficiently far apart to admit the air. Otherwise the drilling of a few holes in each division answers the purpose.

A particularly nice little locker, of a simpler description than the one just mentioned, is in vogue at the Poplar Hospital for Accidents. Its special feature is the open shelf next to the bed, allowing the patient to reach his possessions without any exertion or stretching out of bed. The lockers are 3 ft. 6 in. in height, so that the top, which is of marble, is at a comfortable level for the patient. It is made in polished wood, and has good castors. These latter are specially necessary, in order that there shall be no accumulation of dust beneath, and that the locker may be moved for sweeping purposes easily and without injury to the floor.

The second illustration shows a locker in use at the Great Northern Central Hospital. This is also of teak, and is roomy, and well made. It is intended to serve also as a chair, the lower cupboard being reserved for the patient's own things, and the stores being kept in the cupboard at the back, with a lid which opens from above downwards. The effect is, however, certainly not happy when the lid is down and the stores are displayed to view. There is a towel rail at the back. These lockers are manufactured by Messrs. Atkinson.

All the improvements effected on these points during the last few years must tend to sensibly lighten ward work in many respects, a far greater amount of labour being naturally required to keep in a thoroughly sweet and sanitary condition lockers of the old type, mere cupboards of scrubbed deal, such as are still in use at those institutions where funds have not yet permitted of the substitution of an "ideal locker," which will no doubt be universally accepted within a measurable distance of time.

## Figures and Tables

**Figure f1:**
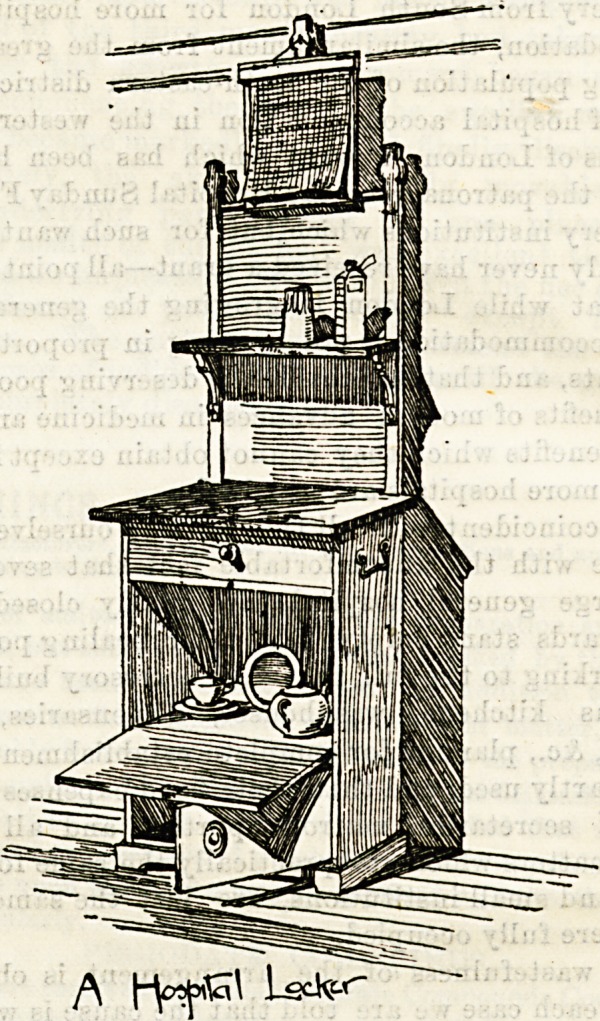


**Figure f2:**